# Editorial: The heart team version 2.0: Optimized approach in 2020

**DOI:** 10.3389/fcvm.2022.1114599

**Published:** 2022-12-15

**Authors:** Livia Luciana Gheorghe, Tanja Rudolph, Sabine Bleiziffer, Ole De Backer, Martin Swaans

**Affiliations:** ^1^Interventional Cardiology Unit, Hospital Universitario Puerta del Mar, Cadiz, Spain; ^2^Klinik für Thorax- und Kardiovaskularchirurgie, Herz- und Diabeteszentrum NRW, Bad Oeynhausen, Germany; ^3^Clinic for Thoracic and Cardiovascular Surgery, Herz- und Diabeteszentrum NRW, Ruhr-Universität Bochum, Bochum, Germany; ^4^Department of Cardiology, Rigshospitalet, Copenhagen, Denmark; ^5^Cardiac Imaging Unit, St. Antonius Hospital, Nieuwegein, Netherlands

**Keywords:** tricuspid valve, multimodality imaging, echo, MDCT, fusion, MRI, tricuspid interventions, TAVI

Severe aortic stenosis and tricuspid regurgitation are both very prevalent valve pathologies. For decades, surgical aortic valve replacement has been considered the standard treatment for symptomatic severe aortic stenosis whereas tricuspid regurgitation was often left untreated. This approach resulted in a tremendous undertreatment, in particular of patients at high surgical risk or with advanced age.

In the past 20 years, there has been an exponential increase in percutaneous techniques for valvular heart disease to overcome these unmet clinical needs. Following this (r)evolution, also cardiac imaging has tremendously developed and nowadays features high-resolution and multi-modality imaging.

Severe aortic stenosis was one of the first valve diseases percutaneously treated and transcatheter aortic valve implantation (TAVR) has become a routine treatment option receiving a class I indication in the latest ESC guidelines for patients over 75 years of age with severe aortic stenosis regardless of their surgical risk.

This series of articles takes into consideration important aspects such as patient-tailored aortic valve replacement and challenging anatomies for TAVR, management of concomitant coronary artery disease before and after TAVR, and also evaluation of patient-prosthesis mismatch (both in TAVR and SAVR).

In the article by De Backer et al., the possible advantages of SAVR vs. TAVR are discussed, thereby highlighting the importance of a careful evaluation, not only for operative risk, but also anatomy, lifetime management and specific co-morbidities in an appropriate Heart Team meeting. Using the latest TAVR generation devices, it has been possible to successfully treat prohibitive anatomies such as bicuspid aortic valve, low coronary ostia and big aortic annuli. However, the review also highlights the possible surgical options, especially in patients with associated aortopathy.

The article by Saad et al. focuses on those challenging anatomies which were, until recently, considered relative contraindications for TAVR. In current practice, outcomes of TAVR in bicuspid or tricuspid aortic stenosis are nearly comparable due to improvement of the devices, implantation techniques and better annulus sizing. Moreover, the review offers a general view of the percutaneous treatment of pure aortic regurgitation and the available non-dedicated and dedicated devices to treat this pathology. Another important topic is the treatment of the coronary artery disease: how to evaluate intermediate coronary lesions, the proper moment to revascularize and the possible challenge to access the coronary arteries once the TAVI device is in place.

Sabbah et al. gives an overview on the role of invasive and non-invasive coronary imaging to assess the coronary arteries prior to TAVR. It is also discussed why increased microvascular resistance due to extravascular compression caused by high ventricular pressures can be a pitfall for the accurate measurement of fractional flow reserve (FFR). Moreover, the review provides the latest evidence for when, and how to revascularize patients undergoing TAVR. Treatment of distal coronary artery disease does not improve TAVR outcomes and most likely only increases the risk of bleeding. Nowadays, coronary revascularization is more related to worries about coronary access after TAVR and less to the concern of possible hemodynamic instability during TAVR.

Selective cannulation of the coronary ostia may be very challenging and depends on patient anatomy, valve type and design, and implantation characteristics such as implantation depth and the orientation of the commissural posts in relation to the ostia.

Weferling at al. comes with recommendations on how to engage the catheter to the coronary ostia and also comments on valve type-specific peculiarities, further discussing and emphasizing the importance of commissural alignment.

Next, another important AVR issue is discussed, namely patient prosthesis mismatch (PPM). PPM proved to be an independent factor leading to worse outcome after SAVR, and multiple studies support TAVR instead of SAVR in patients with small annulus. Bleiziffer and Rudolph discusses the PPM definition and presents some tips on how to accurately measure the indexed effective orifice area (iEOA) after SAVR or TAVR and the impact of PPM on patient outcomes. Importantly, there are used different cut-off values for patients with a BMI ≥ 30 kg/m^2^, being lower than that in normal weight patients (thresholds of 0.65 and 0.85 cm^2^/m^2^ for severe and moderate mismatch in patients with an average weight vs. 0.55 and 0.7 cm^2^/m^2^ for adipose patients).

Finally, this series of articles also discusses treatment options for the tricuspid valve, which was neglected for decades due to silent symptomatology. Surgical treatment (tricuspid annuloplasty in most cases) is the standard treatment of severe-to-torrential tricuspid regurgitation. However, in reality, only a small percentage of patients receive this treatment, with a relatively high mortality rate. Percutaneous treatment has emerged as a new possible therapeutic option for patients with severe symptomatic tricuspid regurgitation and high surgical risk. Moreover, there are different technologies in development to treat this group of patients.

Over the past few years, cardiac imagers, interventional cardiologists and cardiac surgeons combined their knowledge to better understand the anatomy, pathophysiology and the possible results after minimal invasive treatment. The two principal options of treatment are either percutaneous tricuspid annuloplasty or transcatheter edge-to-edge repair (TEER) treatment. Cardiac imaging is absolutely indispensable from diagnosis, over transcatheter treatment up to clinical follow-up. The state-of-the-art review by Wunderlich et al. offers a comprehensive overview of the tricuspid valve anatomy and the relation with the surrounding structures that may interfere during percutaneous treatment. Moreover, percutaneous tricuspid annuloplasty using Cardioband device is meticulously described. Echocardiography and cardiac computer tomography are the main tools used to evaluate the tricuspid valve anatomy. However, intra-procedurally, additional landmarks are needed since the transesophageal echocardiography (TEE) may have limitations due to the anterior position of the tricuspid valve or shadowing. This review reveals some useful fluoroscopy views, which may assist with the positioning of the TEE probe to obtain specific views during transcatheter tricuspid valve procedures.

Finally, one of the unanswered questions is the correct timing for the treatment of severe tricuspid regurgitation. Gheorghe et al. also gives an overview on the current therapeutic options of severe tricuspid regurgitation and the importance of proper hemodynamic evaluation prior to treatment decision. The procedural steps of TEER of the tricuspid regurgitation are also nicely illustrated. Moreover, this review focuses on the multimodality imaging possibilities of the tricuspid valve discussing the (dis)advantages of each imaging technique during tricuspid regurgitation treatment and mentions the potential advantages of the new imaging tools such as fusion imaging and artificial intelligence in this field. A strong multidisciplinary approach made the development of percutaneous options possible providing a beneficial therapy for patients being left untreated in the past.

## Author contributions

LG, TR, and OD: writing. TR, SB, OD, and MS: review the article and correct it. All authors contributed to the article and approved the submitted version.

